# Bioinspiration: something for everyone

**DOI:** 10.1098/rsfs.2015.0031

**Published:** 2015-08-06

**Authors:** George M. Whitesides

**Affiliations:** Department of Chemistry and Chemical Biology, Harvard University, Cambridge, MA 02138, USA

**Keywords:** bioinspiration, chemistry, chemical biology

## Abstract

‘Bioinspiration’—using phenomena in biology to stimulate research in non-biological science and technology—is a strategy that suggests new areas for research. Beyond its potential to nucleate new ideas, bioinspiration has two other interesting characteristics. It can suggest subjects in research that are relatively simple technically; it can also lead to areas in which results can lead to useful function more directly than some of the more familiar areas now fashionable in chemistry. Bioinspired research thus has the potential to be accessible to laboratories that have limited resources, to offer routes to new and useful function, and to bridge differences in technical and cultural interactions of different geographical regions.

## 1. Introduction

### 1.1. Where do ideas originate?

In science and technology, ideas often come from studying Nature [1,2]. Kepler and Newton developed the first empirical descriptions of gravity by studying the motions of the Sun and the planets. Faraday and Maxwell derived the fundamentals of electromagnetism by examining interactions between electrical currents and magnets. Thermodynamics was derived from studies of heat transfer and mechanical work. Quantum mechanics originated, in part, from spectroscopic studies of light. The current objects of attention in *chemistry*? There are, of course, many, but probably the most abundant of them is biology, and its study has tended to focus on the molecular basis of genetics, the characteristics of cells, the development of higher animals and disease.

### 1.2. Biology, and bioinspiration, bioimitation and biomimicry

**Figure 1.**
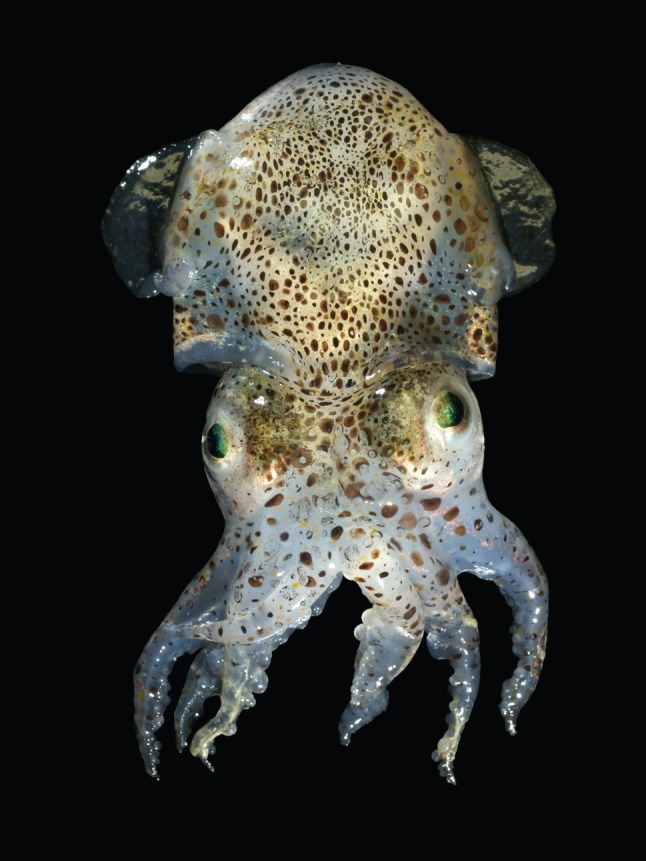
Nature is rich with sources of inspiration for new ideas. ‘Biomimicry’ aims to mimic or recreate some of the interesting properties of biological systems in non-living ones. Consider the squid: we need not understand how a tentacle moves to recreate at least some of its motion. Photograph by Hans Hillewaert.

Biology—the study of life, from single cells to complex organisms—is one set of subjects. It leads, however, to a rich mixture of others that are not exclusively focused on living systems, and one of these—one a bit to the periphery of the torrent of information emerging from molecular biology—might be called ‘biomimicry’ or ‘bioimitation’. The objective of this subject is to mimic or imitate characteristics of biological systems in non-living systems, rather than replicating or analysing the biological entity itself, in molecular detail. As an example, the processes—the combination of systems of sensors, muscles and brain (and other organs that process information)—that allow a squid to control its tentacles are still beyond us (figure 1). Understanding enough of the mechanics of a tentacle to *mimic* some of its characteristics, even if the mechanisms used in that mimicry are unrelated to those used by the squid, is a simpler, more immediate and perhaps more useful activity.

Understanding the squid (or any living creature) in molecular-level detail is a subject of such multilayered difficulty that it will probably occupy much of science for the next century. Abstracting simplified versions of squid-like behaviour—that is, taking inspiration from its capabilities, and mimicking some of its functionality, but using simplified and probably different mechanisms—is a related, but distinct endeavour with enormous appeal, and with the potential both to stimulate the invention of new processes (i.e. processes found neither in squid nor, at present, in synthetic systems), and to use these processes to solve problems that a purely biological solution would not be able to solve.

### 1.3. Imitation? Abstraction and reuse? A mashup? Motivation?

Is the abstraction and simplification of biology a less demanding, and less rewarding, activity than studying biology itself? It is certainly less demanding: living systems are, without exception, extraordinarily complicated. Even the simplest unicellular organisms—after decades of study—continue to show properties and processes that we can neither understand nor duplicate. That ultimate complexity notwithstanding, an understanding of the rudiments of biological function (e.g. higher-level phenotypic behaviours) allows us to begin to appreciate processes of optimization of function that have appeared over billions of years of Darwinian evolution. This appreciation clarifies why cells and organisms have the structures that they do, and provides shortcuts to ‘lifelike’ function in synthetic systems. To mimic a function, we need not understand it completely. We do not need to understand how solvents work at the molecular level to use them in organic synthesis. An aeroplane does not use the same processes as a bird to fly, but bird and aeroplane share the idea of flight. A methane–air flame and a cell both ‘burn’ a reduced form of carbon (methane or glucose); the two processes, however, produce distinctly different outcomes, and these differences are a source of great inspiration to those interested in understanding the cell as a dissipative system. Biology stimulates new ideas about function and its origins.

Even if the detailed mechanisms that make them happen are not fully understood, the marvellous phenomena that characterize biological systems can sometimes be imitated, abstracted and patched together to provide a rich set of scientific and technological puzzles, many of which are starting points for invention. When using biological systems as a source of behaviours and functions to imitate, there is no single ‘correct’ pathway: biology provides essentially endless stimulating illustrations of successful designs, and we are free to use them as we will.

## 2. Biological systems and bioinspiration

### 2.1. Bioinspiration, molecules, materials, structures and functions

Because biology offers remarkable examples of new structures and processes at every scale, where should one focus? In biology, at all scales, *function* is the key idea: organisms cannot afford—in the great Darwinian competition—to decorate themselves with functionless features. By trying to mimic the behaviours and properties of living systems, we are therefore automatically engaged in mimicking functional processes and structures. ‘Bioinspiration’ thus leads—directly or indirectly—to function, some of which is useful to the organism, and some of which *might* be useful to us. Surrounding *function*, however, are other, more conceptual ideas that guide the selection of problems—and the design of appropriate research strategies—in bioinspired investigations.

### 2.2. Some characteristics of biology and bioinspiration

We have found three intellectual vectors to be particularly interesting and useful in choosing characteristics of biological systems to try to understand and *imitate*.

#### 2.2.1. Function

Organisms seldom waste energy on the generation of structures that serve no function. They cannot afford to: they will be eaten, unless they focus on characteristics that give them an advantage. We often do not understand what that advantage might be, or how it is achieved; but both questions are rewarding to study, even if they do not lead to functions of immediate value to us [3]. Still, much of technology is ultimately about function (almost no one cares about the molecular structure of polystyrene, only that it is one of several compositions of matter from which one can inexpensively fabricate coffee cups and similar useful objects). By studying function in biology, one starts close to a functional end.

#### 2.2.2. Simplicity

**Figure 2.**
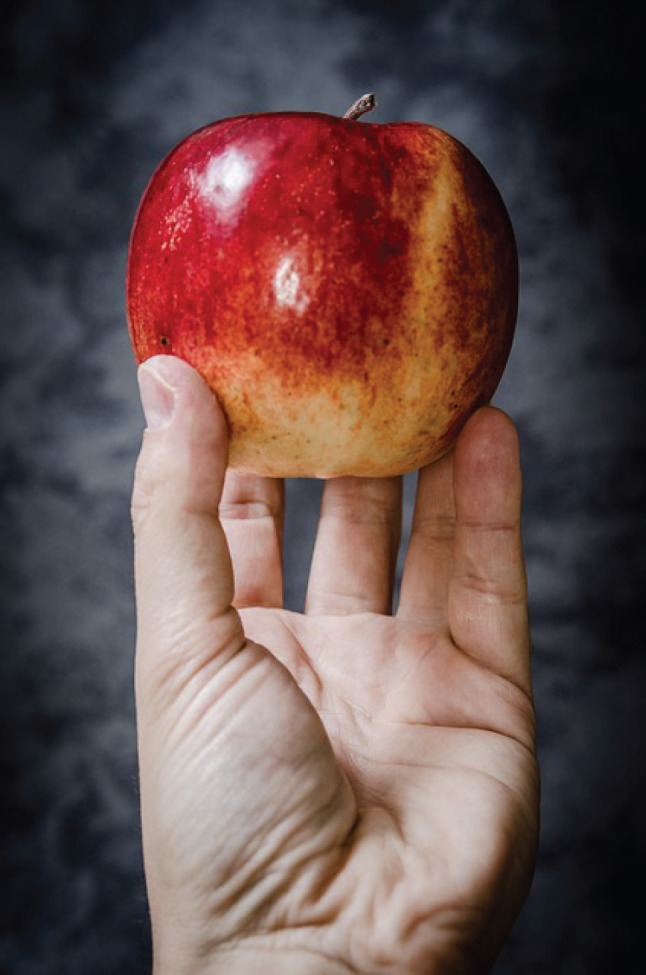
Biology seamlessly blends chemical and mechanical systems to make complex tasks—such as picking up an apple, and eating it—seem elegantly simple. Biological systems thus have an enormous amount to teach about the way in which complex subsystems combine to yield simple, reliable, functions.

Many biological systems are marked by elegance in design; complex mechanisms and structures blend to become apparently simple functions*.* Picking up an apple, and eating it, seems so functionally simple that it barely warrants thought. Examined in detail, however, picking up and eating an apple consists of a network of systems of extraordinary complexity, operating in sequence and in parallel, often almost invisibly (figure 2). Biology has an enormous amount to teach about the integration of complex subsystems into simple, reliable, functions.

#### 2.2.3. Dissipation

Almost all interesting biological systems are dissipative: that is, they function only when there is a flow of free energy through them. Much of chemistry and materials science is still frozen in a non-dissipative world. A polystyrene cup is not at equilibrium with its environment, but it is at stationary state: its function does not depend upon a flow of energy through it. A frontier of science is (in my opinion, and in the opinions of many others) the study of dissipative systems and structures (organisms, cities, weather patterns, traffic, financial markets) [4,5]; one cannot understand dissipative systems without, ultimately, studying (and being inspired by) life.

### 2.3. Scientific ‘impact’

Another motivation to understand and mimic biological systems has to do with a subject on which scientists have very different opinions. One (although not the only) reason to do research is to catch the curiosity of other scientists, to contribute to their research and, perhaps, to encourage them to try new ideas (i.e. to have an ‘impact’ on their activities, and ultimately, through them, to contribute to solving problems important to society). Because almost everyone is interested in living things, and because the path from observation to a successful scientific or technological outcome may be shorter in bioinspired research than in other kinds of research, bioinspiration has historically been a rewarding area in which to work for those interested in scientific impact.

### 2.4. Soft matter

In even broader terms, bioinspiration leads directly to a field of science that is now growing rapidly in importance and interest: that is, the science—especially the materials science—of soft matter [6]. Materials science grew up around structures that were ‘hard’ (durable, fixed in shape and function, resilient to damage). Most biological systems (even including, at some level, bone) are ‘soft’: i.e. elastic and easily deformed. This type of matter has been much less explored than ‘hard’ matter, and thus offers opportunities for discovery and invention that are relatively unexplored (in science), and simultaneously both independent of biology and relevant to biology. Understanding the variety of ways in which organisms use soft matter—muscle, tendon, connective tissue, membranes, nerves—offers an enormous range of stimulating ideas for new, soft, science.

## 3. Examples of bioinspired work from our own research

Our research has explored a number of areas in which bioinspiration played an important role. I sketch three in the following.

### 3.1. Self-assembled monolayers

One important characteristic of all biological systems is that they are compartmentalized. For a variety of reasons, organelles, cells, tissues, organs and many other structures are surrounded by membranes or structural films. We—and many others—have been stimulated (‘inspired’) by the remarkable, self-assembled bilayer structures (the prototypical ‘walls' of the compartments that define the dimensions of cells and organelles) that phospholipids form by molecular self-assembly [7]. Rather than working with lipid bilayers themselves, we have worked with the structurally simpler, monolayer films that form on the surface of many metals and metal oxides when they adsorb *n*-alkyl groups with terminal functionalities that interact strongly with their surfaces.

**Figure 3.**
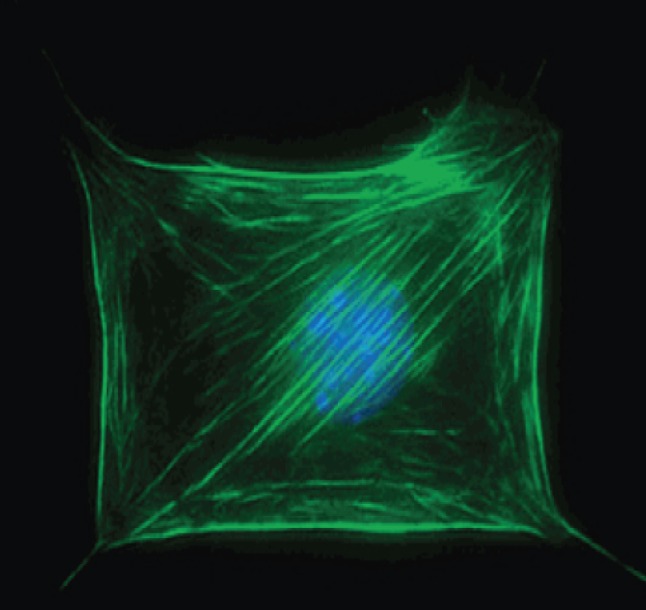
Self-assembled monolayers (SAMs)—monolayers of *n*-alkyl groups adsorbed to the surface of metals and metal oxides—are bioinspired structures that have opened up entirely new avenues of research. SAMs are often used not only to study non-biological functions, but they have also contributed to the understanding of interactions between cells and the surfaces to which they are attached. Reproduced with permission from [9]. Copyright © 2003 American Chemical Society.

The chemistry of these types of systems—that is, of self-assembled monolayers (SAMs)—is, by now, familiar [8]. One of the characteristics that makes them particularly interesting, in the context of bioinspiration, is that they are based on ideas about forming complex organic structures (e.g. self-assembly and non-covalent synthesis) that are ubiquitous throughout biology, but non-trivially countercultural in a world of organic synthesis that has evolved around the assumption that ‘synthesis' should principally focus on stepwise formation of *covalent* bonds in molecularly complicated but, nonetheless, simple (relative, say, to a cell membrane, a protein or other cellular structures) entities. The fact that self-assembly and non-covalent structure are ubiquitous throughout biological systems suggests that they are valuable as the basis for another kind of synthetic strategy. Additionally, and importantly, biology teaches—and SAMs and other bioinspired structures confirm—that self-assembly can generate (easily and practically) structures much larger than those that can be generated by classical covalent synthesis, and thereby provide a bridge between molecules and macroscopic matter. SAMs were among the early examples of ‘materials by design’—that is, materials in which variations in the structures of easily synthesized, low-molecular-weight building-blocks yield control over the structure and functionality of macroscopic areas of a material (i.e. areas of 1–100 cm^2^ composed of 10^14^ to 10^16^ individual molecules). We have used these structures primarily to study non-biological functions, although they have contributed to an understanding of the nature of interactions between cells and surfaces (figure 3) [10]. SAMs have become standard substrates in surface science for the study of wetting, adhesion, lubrication and charge tunnelling [11–13]. Because of the simplicity with which they can be assembled, and the exceptional ease with which their molecular-level structure can be controlled, the impact of SAMs on surface science has been high. All these ideas were, of course, developed and illustrated in biology long before surface science and materials science existed; they provide one example of ‘bioinspiration’.

### 3.2. Microfluidics and paper diagnostics

**Figure 4.**
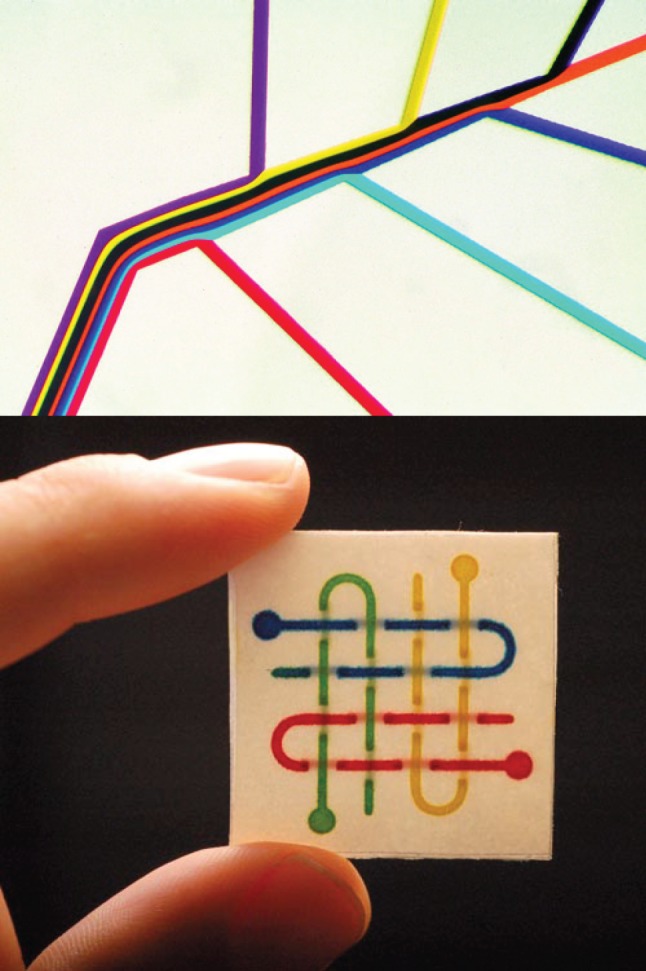
Micrometre-sized microfluidic channels mimic the capillaries through which fluids flow in living organisms. These channels, which are easy to fabricate via methods that evolved from the materials and processes developed for patterning self-assembled monolayers, are useful for the study of biology, and as the basis of diagnostic devices.

All but the smallest organisms must supplement the passive diffusion of molecules with active pumping of fluid to achieve rates of biomolecular transport necessary for survival and propagation [14]. In large animals, fluid is pumped through arteries and veins, connected by capillaries. An interest in the properties of fluids in small channels was one stimulus for the development of microfluidics—the movement of fluids in channels having dimensions similar to those of capillaries (figure 4) [15,16]. We have been actively involved in the development of microfluidic systems (primarily for their potential value in diagnostic systems [17,18], rather than in biology); the fabrication methodologies that we developed were, in fact, derived directly from materials and processes that we developed for patterning SAMs by stamping. These methods—largely based on polydimethylsiloxane (PDMS) slabs whose surfaces presented moulded topographical features (typically between 100 nm and 100 μm)—became the basis of microcontact printing, micromoulding, much early work on microfluidics and, ultimately, the foundation of ‘soft lithography’ [19].

Although the motivation of this work was partially biomimicry, it inspired a wide range of research only distantly related to biological function. The most active of these research areas has been the development of systems of microchannels as the basis for new classes of bioanalytical and diagnostic systems (the ‘lab-on-a-chip’ systems originally imagined by Dittrich & Manz [20]). The applications for elastomer-based microfluidic systems are still being developed—for bioanalytical systems, for diagnostics and as a part of emerging areas such as ‘organ-on-a-chip’ systems [21]. To the point of this article, however, PDMS-based microfluidics used ideas inspired—at least, in part—by the circulation of fluids in larger organisms, and nucleated a new area of analytical chemistry and fluid physics, which continues to prosper. The structural similarity of microfluidic channels to biological microchannels, in addition to their ease of fabrication, provides ideas for their development, guidance in understanding their supported fluid flows, and assurance that they can be fabricated in almost any laboratory. (Although PDMS is not universally available, many other elastomers—including food gels—can be used to make microfluidic systems.) Bioinspiration thus contributed important (and critically, *simple*) ideas to a new field of bioanalytical chemistry, materials science and fluid physics.

### 3.3. Soft robotics

**Figure 5.**
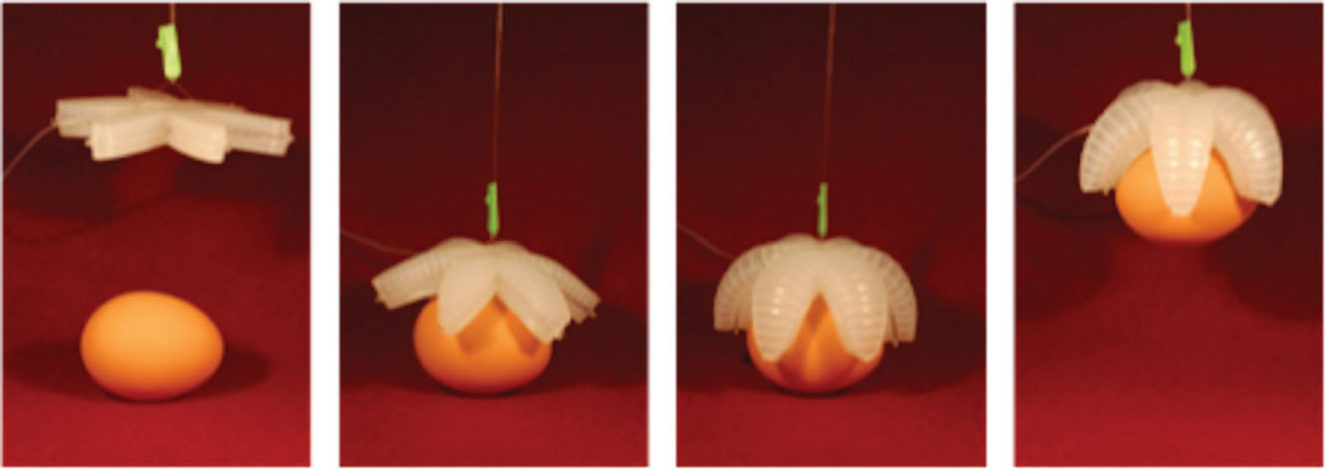
A starfish may not use its appendages to grip objects, but its form inspires mechanical structures that do. Pneumatically actuated soft robotic grippers, which resemble the shape of a starfish, can gently pick up and release an uncooked egg and other fragile, irregularly shaped objects with ease [22].

Remarkably, the methods developed for microfluidic systems could be used, almost without modification, in another area with a strong basis in bioinspiration: that is, soft robotics (figure 5) [22,23]. The interest in this area is with structures with much larger dimensions than those important for SAMs and microfluidics. Two observations served as the starting inspiration for this now rapidly developing field. (i) Robotics based on mimicking and augmenting the motions and characteristics of large mammals had become a well-established and important field (especially in manufacturing). These robots were typically fabricated in metals, and actuated electrically or hydraulically; they were designed to accomplish tasks that required large forces and high speeds; they were heavy and expensive; they were dangerous for humans. (ii) Most of the organisms found on Earth are, of course, not large mammals, and do not have the body plans of dogs or humans; they are, instead, soft: worms, squid, spiders and most of the other, most prevalent forms of life. Our initial efforts in soft robotics were based loosely on the inspiration provided by starfish, and were designed to make simple grippers. The mechanism of actuation in these ‘starfish grippers' involved pneumatically inflated networks of microchannels embedded in PDMS (‘PneuNets'), designed so that inflation resulted in anisotropic motion [24].

**Figure 6.**
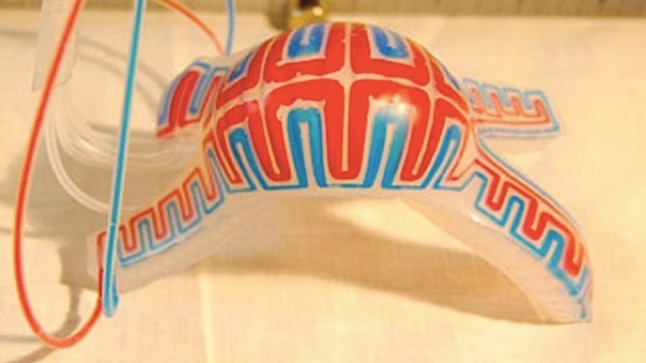
Cephalopods such as squid, octopuses, and cuttlefish use their ability to change colour to hide from predators. This soft robot uses simple microfluidic channels to camouflage its ‘skin’, hiding from detection in both the visible and infrared [23].

The area of pneumatically actuated soft robots has grown extremely rapidly, in part because the methods required to make soft structures were well developed for PDMS-based microfluidics, and in part because biology provides so many examples of soft organisms, and so many examples of motions, that many interesting natural actuators and structures are available to imitate (figure 6). In biological systems, of course, actuation is ultimately based on muscle (and on structures requiring muscle, such as hydrostats). Soft robots, thus, may mimic some of the motion and function of biological systems, while using none of the mechanisms involved. Mechanism is, however, at a certain level irrelevant: what has made the field grow so rapidly is the ability to mimic function (not mechanism).

## 4. The future

### 4.1. Limitless opportunities

The enormous range of functional solutions to the many problems faced by living organisms guarantees that ‘bioinspiration’ is a field that will continue to provide excellent science for many decades. At almost all levels of complexity and function—from ‘simple’ structures such as seashells (which are extraordinarily sophisticated heterogeneous composites designed to dissipate the energy of attackers trying to breach the shell) to the (currently) unassailably difficult problems of sentience and memory—biology provides examples of function at every level of complexity. Let me offer a few examples of problems of different types that can be explored, now and in the future.

### 4.2. Molecular-level phenomena

At small dimensions, biology provides a series of examples of molecules, molecular aggregates and supramolecular structures of sophistication in function that science has so far not come close to imitating. Four (of many) examples include ‘motors’, muscle, cell membranes and enzymatic catalysts [25]. (i) *Molecular aggregates with rotary motions.* One of the distinctions between biological and human-made systems is that *wheels* are ubiquitous in human-designed machines, and biology uses them in only a few (very important) circumstances [26]. We use wheels everywhere, from trucks to watches. In biology, rotary motors drive the flagella that allow microorganisms to swim. Remarkably, structurally similar aggregates are used in all cells to pump ions across membranes, and to use transmembrane concentration gradients of ions to synthesize adenosine triphosphate (ATP). These biological structures provide a demonstration-of-principle of the idea that it is possible to achieve functional rotary motion on the molecular scale, especially using nanoscale structures embedded in cell membranes. Mimicking such systems on the molecular scale presently seems unachievably difficult, but it provides one example of a grand challenge. Ben Feringa has provided one elegant small-molecule mimic of motion of this type, and thus has taken a creative first step towards more complicated systems [27]. (ii) *Muscle.* Muscle has a similar structure across an astonishingly broad range of organisms. Aggregates of two fundamental proteins—myosin and actin—form the basis for several types of structures that ‘walk’ past one another, and the resulting motion can do work; the free energy required is provided by hydrolysis of ATP. We have no models for these types of systems at any level of complexity. (iii) *Multifunctional cell membranes.* Biological membranes are typically simplified conceptually as quasi-two-dimensional, self-assembled structures formed by self-assembly of surfactants (e.g. phospholipids) [3,28]. They are often depicted as regular, ordered sheets studded with occasional glycoproteins and glycolipids. In fact, a more accurate, functional description might describe them as quasi-two-dimensional aggregates of proteins and oligosaccharides, with lipids filling the cracks between these functional entities. That is, they *do* provide boundaries to cells and organelles, but more interestingly, they provide complex aggregates of mobile and heterogeneous components with catalytic, recognition and signalling functions. Whichever description one uses, the range of functions that biological membranes serve—selective or passive transport, active recognition and pumping, energy storage, information transfer, signalling and so on—remain to be explored in bioinspired systems. (iv) *Catalysts: enzymes and ribozymes.* In biology, both metabolism and information processing are accomplished and controlled by molecular catalysts. Enzymes, as the most diverse of these classes of catalytic structures in biology, are often thought of as analogues of more familiar chemical catalysts (protons, platinum surfaces, soluble transition metal complexes and so on). In fact, they are more than simple catalysts. One of the most remarkable characteristics of enzymes—and one critical for their function in the cell—is that their catalytic activity is modulated by other molecules in the cell, including their own reactants and products; this modulation is a key part of the metabolic networks on which the cell depends for its internal regulation, and central to the activity of the cell throughout the cell cycle. Enzymes are also shape-selective, and thus a key part of maintaining the integrity of molecular selectivity of reaction networks. Of course, they *are* catalysts. Chemists have become reasonably skilled at building certain types of catalysts that show shape selectivity (although usually not with the selectivity of enzymatic catalysts); they are not skilled at designing new classes of catalysts de novo; and there are very few examples of synthetic catalysts that show the modulated activity of enzymatic systems. Catalysis is critical for life; catalysis is also critical for chemical processing. There is an obvious mutuality of interest between biochemists, synthetic chemists and biomimetic chemists in the design of catalysts that work in aqueous solution, and (more importantly) that form networks in which the components communicate among themselves through their responses to the molecules generated and processed by these networks. Studying biological catalysis, with the objective of understanding principles that could be embedded in non-biological catalysts, is an important opportunity.

### 4.3. Meso- and macroscale structures and phenomena

Throughout the animal kingdom, there are countless examples of actuators (e.g. arms, legs, wings, tentacles and fins), each with unique advantages for some environment and set of tasks, and each with lessons to teach those interested enough to watch, and to try to imitate [3]. I offer a few examples. (i) *Body plans.* The much despised cockroach is a remarkably versatile insect. It is capable of making its way over extraordinarily rough terrain, of gliding through small cracks and of changing its gait (e.g. sprinting on two legs). It is an ideal insect for inspiring devices for use in search and rescue, and surveillance in difficult environments. The kangaroo offers another example: its ability to store energy in the tendons of its hind legs gives it the ability to navigate certain types of terrain with extraordinary speed and agility. For that environment, it is an exceptionally energy-efficient model of locomotion. The gecko, another example still, has setae-covered feet that make it uniquely well equipped to climb walls [29]. (ii) *Sensor systems: eye, nose, ear and others.* Sensors of all sorts are critically important to animals: in locating food, discerning danger, mapping an environment. There have been a wide variety of attempts to imitate the properties of biological sensors. The so-called electronic dog's nose, for example, substitutes the solubility of odorants in polymer matrices for shape-selective molecular recognition by receptors [30]; such biomimetic systems work, but only to a limited extent. The enormous range of ‘eyes' that have evolved in biology has been more difficult to imitate than the nose: we have, based on semiconductor technology, a highly developed ability to generate planar focal plane arrays; the curved arrays of rods and cones that provide sensing to the retina, however, have proven more difficult to fabricate, and the advantages of compound eyes, mammalian eyes and the eyes of squid and others are poorly understood [31]. ‘Touch’ is being actively explored in electronic systems based on capacitive sensing in polymeric matrices [32–34]. The abilities to model more complex functions—balance, proprioception, echolocation (in bats and porpoises), high-sensitivity IR (e.g. heat) detection (in pit vipers)—all remain as marvellous, unsolved challenges. (iii) *Colour and camouflage.* Colour—for a wide range of purposes, from display to camouflage—is also critical to animals. Pigments we understand; optical metamaterials (as in the morpho butterfly) are currently a subject of intense interest; the almost unbelievable ability of the mimic octopus both to sense the coloration of its environment, and to mimic it, remains an inspiration for future work [23,35].

### 4.4. Information

Information—and its collection, storage, processing and interpretation—is at the heart of our society [36]. New ways of looking at information will likely find surprising applications. Biology uses many strategies to process information that do not conform neatly to the binary world pioneered by Shannon [37] and others (and realized in semiconductor technologies). We have largely passed by analogue computation in favour of digital computation, for reasons that are generally excellent when the problem can be converted efficiently to manipulating bits. Biology has largely passed by digital computation in favour of analogue, also for excellent reasons. Imitating strategies used in living organisms to store and process information provides an exceptionally interesting, rich and virtually unexplored area for bioinspired research.

### 4.5. Use of energy

Many biological systems are substantially more energy efficient than analogous synthetic systems that are, in a sense, biomimetics [38]. A pony-sized hard robot, for example, uses about 100 times more energy than a pony, to do fewer functions. Why? We do not understand the constraints to efficiency in biological systems in the detail we understand the thermodynamics of work carried out by mechanical, human-made heat engines. Relative weight is one contributor to the difference (metal is more dense than tissue), but there are many; organisms have diverse strategies for storing and regenerating energy; actuation proceeds in different ways in biology and in mechanical systems; the requirements for power and durability impose different constraints on biological and human-made systems. Looking at organisms from a thermodynamic perspective, and understanding—at the simplest level—the basis for their ability to do mechanical work, is still an immature area of science, and drawing lessons from organisms to build more functional and efficient machines is earlier still [39]. Because efficient use of energy is so important in almost all applications, it is a subject of great practical use.

### 4.6. Reaction networks

One of the remarkable characteristics of cells—as the ultimate, living, biological construct—is their ability to construct and manage extraordinarily complicated networks of synthetic reactions (e.g. metabolism and replication) [40]. These networks are like nothing we can rationally construct. A key to their operation seems to be the ability of catalysts that are ubiquitous in cells (in the form of enzymes and ribozymes) to have their activities modulated by the starting materials and products of other key reactions [41,42]. The central element that underlies modulation is, thus, the ability of individual reactions to ‘talk to one another’ through environmentally sensitive catalysts (especially enzymes) and to maintain a stable, dissipative, non-equilibrium state over many cycles of cell division. How do these networks work, and why are they stable? (This question is, in a sense, another way of stating the question ‘What is life?’) Understanding, and imitating, the complex kinetic networks of metabolism is an extraordinarily ambitious objective for bioinspired research.

### 4.7. Covalent and non-covalent synthesis: molecular self-assembly and water

An example of an apparently simple set of molecular processes—but one at the very core of biology—is molecular recognition and non-covalent self-assembly. Synthetic organic chemistry has developed an extraordinarily complicated set of empirical methodologies for manipulating covalent bonds. Biology, also, of course, synthesizes covalent bonds, but the most important processes (the formation of phospholipid membranes, the folding of proteins, the pairing of bases in DNA and RNA, the association of proteins with substrates, ions and signalling molecules) generally involve non-covalent chemistry. Remarkably, we understand very little about molecular recognition in aqueous solution, and almost nothing about molecular recognition in the much more complex environment of the cytosol. Molecular recognition has been extensively examined in non-aqueous solvents, using simple (for example, hydrogen bonding) interactions. The much more complex set of ionic, hydrogen bonding and dispersive interactions involved in molecular recognition in water and water-based media is much more poorly understood [43]. Understanding molecular recognition in water, and being able to mimic the specificity of biological systems is a problem with enormous practical implications for areas ranging from rational drug design to water purification.

## 5. ‘Bioinspiration’ as a subject that bridges geographical and technological differences

### 5.1. Imitation in styles of science

Academic chemistry developed in Europe and the USA, and it has tended to favour research carried out at the extremes of complexity (targets for synthesis are based on organic structures chosen for the aesthetics of their complexity; targets for spectroscopy require ultra-fast and ultra-high-power lasers; targets for protein structure analysis in solution are limited, *inter alia*, by the state of technology involving very high magnetic fields). It is difficult for laboratories (academic or other) that do not have abundant resources to compete in many of these fields of research, in a style where ‘most extreme’ often translates as ‘most complicated and most expensive’. This focus on complicated science has the unfortunate characteristic that it can seem to exclude scientists working in technologically less well-developed laboratories from the newest areas of research. One, thus, sometimes finds laboratories—especially in the scientific systems of the developing world—imitating structures, styles and objectives that were current in European and American science 50 years ago.

An attraction of bioinspiration as a strategy in research is that it is, at its core, a relatively simple one. The organism or system that offers remarkable new phenomena for study may not require an electron microscope to observe, but may, instead, be eating birdseed from the hand of the investigator; targets of interest (muscle-like actuation, acoustic systems for underwater echolocation, tentacle mimics and countless others) may be—at the level required for bioinspired research—already well defined through centuries of human observation. Bioimitation is usually best accomplished by the simplest strategy, rather than by using the most complex instrumentation. Deep familiarity with biological subjects may actually be greater in regions in which scientists spend more time out-of-doors, observing nature, than indoors, observing instrumentation. So, in many areas of bioinspired science (as with our programme in soft robotics), there is nothing that could not be done as well in a relatively simply equipped laboratory as in a much more expensively equipped one.

### 5.2. ‘Understanding’ combined with ‘use’. Science, engineering and Pasteur's quadrant

**Figure 7.**
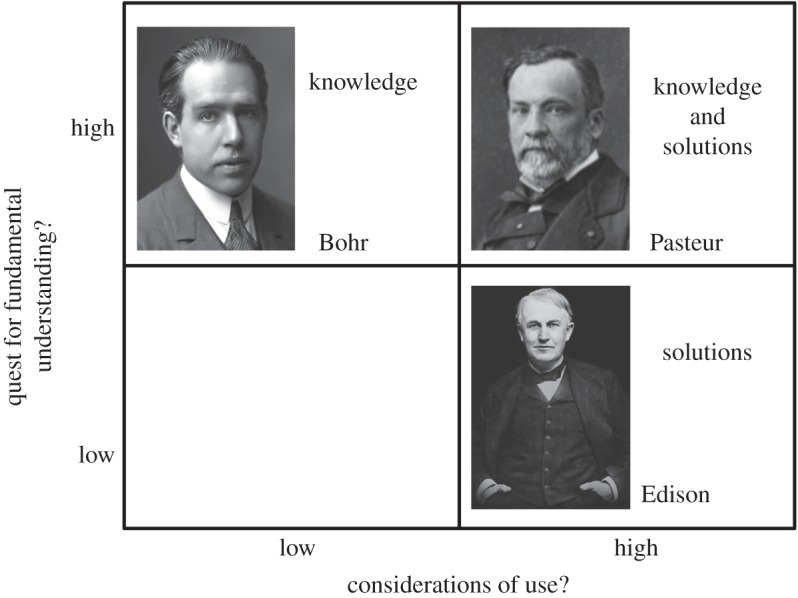
The quadrant diagram, originally proposed by Don Stokes, summarizes his perspective on different styles of research [44]. Bioinspired research lies, almost by definition, in the fourth quadrant (Pasteur's quadrant), where fundamental scientific understanding is closely linked with technology aimed at impacting society. Adapted from [44].

Bioinspiration as a strategy in research offers another interesting opportunity to less-well-resourced scientific systems. The European/American academic model has typically focused on scientific papers as its primary output, rather than on the solution of societal problems, or the creation of jobs [44]. Because bioinspiration is based on biology, and biology is ultimately the study of function, the step from bioinspired research to application may, in fact, be smaller than the same step from the subjects of focus in many typical European or American research programmes. (As an example, the interval in time between the publication of the first paper in soft robotics, and establishing a company to design and build soft robots, was only 2 years.) Thus, using the arguments originally introduced by Don Stokes and summarized in his Pasteur's quadrant diagram (figure 7) [45], bioinspired research lies, almost automatically, closer to Pasteur's quadrant—the quadrant that has the potential to combine a problem important to society (e.g. developing human-compatible assistants for unpleasant tasks in healthcare, or generating inexpensive machines for difficult jobs such as fruit picking) with new science (the mechanical behaviour of soft, elastomeric matter at high strains and strain rates).

### 5.3. Observation as the basis for science. Different regions and styles bring different advantages

Bioinspired research, thus, offers an interesting method of rebalancing the advantages of different geographical and cultural regions in scientific and technological discovery. Europe and the United States will continue to have more sophisticated—and more expensive—equipment than laboratories in economically developing regions; this latter group of laboratories may have, however, empirical contact with natural phenomena that is more direct than that of laboratories focused on exercising expensive instrumentation; thus, they will have better instincts about organisms, processes or structures that could provide the basis for new discoveries.

## 6. Conclusion

Bioinspired research is, in a sense, a return to the classical origins of science: it is a field based on observing the remarkable functions that characterize living organisms, and trying to abstract and imitate (or mimic) those functions.

### 6.1. Biology is function

The starting point of bioinspired research is, in fact, quite different from classical chemistry. It is not focused on molecular structure, or on complex synthesis, or on high-resolution spectroscopy; rather, it is based on observing the functions performed by the products of millennia of Darwinian evolution, on understanding the processes that underlie these functions, and then on imitating or mimicking interesting or relevant aspects of these functions without the constraints imposed by biology (a soft robotic gripper need not worry about feeding, defence against predators, replication, breeding and all the other requirements of life). Bioinspired science, thus, steps automatically beyond the current accepted paradigm in chemical science (‘chemistry is about molecules and reactions') and moves to a different kind of chemistry that mixes molecular science, biology, anatomy, physiology, zoology, robotics, separations science, sensing and other areas not always associated with the core scientific problems of chemistry [44].

### 6.2. Bioinspiration is an effectively limitless source of ideas

**Figure 8.**
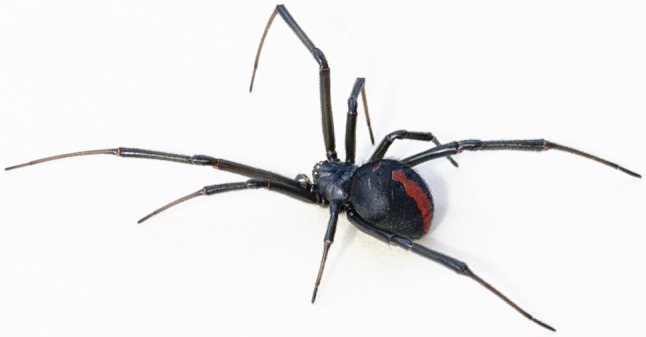
A spider alone can inspire innumerable research programmes: roboticists learn from its gait, materials scientists aim to recreate the strength and structure of its silk, and microscopists study its vision. Photograph by Tony Hudson, CC BY-SA 3.0.

Because there are so many different kinds of organisms, and so many different strategies that have proved successful in biology at solving some functional problem, bioinspiration—as a strategy for developing new ideas—is essentially limitless (figure 8). Experience with biology suggested that almost nothing is simple. High-level functions (e.g. recognizing a face, grazing, swimming) may seem functionally simple, but are supported by layer upon layer of underlying structures, processes and—ultimately—molecules, all interacting with enormous sophistication and complexity. There is no chance that bioinspired research will run out of interesting phenomena to probe.

### 6.3. Simplicity

Often, mimicking a good idea can be much easier than precisely replicating it. We do not have to know how a bird flies to make an aeroplane, or how a squid thinks to make a tentacle. Bioinspiration is still a young field, and good ideas can sometimes be simple.

### 6.4. Bioinspiration is also a trans-cultural field

The fact that bioinspiration returns to observation of nature as a source of problems makes it part of a grand tradition. The fact that many of the most interesting phenomena contributing to the ‘inspiration’ of ‘bioinspiration’ can be observed directly, using no more than the human eye, is so much for the better. The simplicity (when viewed through the lens of a new concept) of many of the problems that emerge from a bioinspired strategy, combined with the fact that different geographical and cultural regions have different types of contact with animals, fish, plants, birds and even microorganisms, means that different regions will have (assuming they exploit them) intrinsic advantages in areas in which their natural landscape is rich. I, for example, know virtually nothing about marsupials, and could not compete with an Australian materials scientist with a zoological bent in thinking about the interesting characteristic of this class of animals. Similarly, I have never seen a wandering albatross, and have no intuition for its astonishingly energy-efficient flight. (I have, however, my own advantages: I have, for example, seen many cockroaches, and I am a great admirer of their agility and adaptability.)

### 6.5. Doing the next thing, not the last thing

Finally, I would remark that although bioinspiration is a solidly established strategy in the chemical sciences, it is not (yet) a central theme in the mainstream. So much the better! Especially in regions that are still developing their scientific and technological systems—both academic and industrial—it is always better to be doing the next thing, rather than the last thing. Bioinspiration offers that opportunity.
